# The association between maternal overprotection and idol worship among college students: the chain mediating role of reactive anger and cybervictimization

**DOI:** 10.3389/fpsyg.2025.1525326

**Published:** 2025-07-02

**Authors:** Yaoyao Li, Xiaogang Wang, Ludan Zhang, Liang Chen

**Affiliations:** ^1^Faculty of Psychology, Shandong Normal University, Shandong Provincial Key Laboratory of Brain Science and Mental Health, Jinan, China; ^2^Law School, The University of Edinburgh, Edinburgh, United Kingdom; ^3^School of Education Science, Guangxi Minzu University, Nanning, China

**Keywords:** maternal overprotection, idol worship, reactive anger, cybervictimization, college students

## Abstract

**Introduction:**

As a unique socio-cultural phenomenon, idol worship plays a significant role in the development of college students' ideological and moral values. This study aims to investigate the influence of maternal overprotection on idol worship and examine the mediating roles of reactive anger and cybervictimization.

**Methods:**

A total of 847 Chinese college students (23.50% were male; Mage = 19.57, SDage = 1.25) were investigated in this cross-sectional survey from 7 to 12 January in 2024 by S-EMBU-C questionnaire, celebrity attitude scale (CAS-R), trait anger scale (TAS), and cybervictimization questionnaire, testing the chain mediating effects of reactive anger, and cybervictimization.

**Results and conclusion:**

(1) Maternal overprotection significantly positively predicted idol worship; (2) Cybervictimization played an independent mediator role in the links between maternal overprotection and idol worship; (3) Reactive anger and cybervictimization played a chain mediating role between maternal overprotection and idol worship. These findings highlighted the association between maternal overprotection and idol worship among college students, highlighting the need for integrated interventions targeting parenting styles, and online safety.

## Introduction

An idol is a product symbolizing the personality of a specific individual. Its image characteristics include beautification, secularization, entertainment, virtualization, etc. These characteristics make the idol no longer represent its own person, but a symbol that can arouse emotional resonance (Song, 2023). Idol worship refers to an individual's appreciation, acceptance, and even imitation of another person's values, behavior patterns, and physical appearance regarding cognitive, emotional, and personality development (Yang and Wu, 2021). Its core issues are psychological identity and emotional attachment (Yue, 2004). In the era of digital new media, with the continuous emergence of variety shows, idol worship has become a common phenomenon and has appeared in more diverse forms (Shi et al., 2016). Furthermore, the demographic scope of idol fandom has expanded significantly, with emerging adulthood (aged 18–25) increasingly constituting the dominant demographic cohort (Lu, 2018). A representative sampling survey revealed that 51.9% of university students exhibit significant idol worship behaviors (Wang and Tan, 2012). As a unique social and cultural phenomenon, idol worship plays a vital role in the development of college students' identity, morality, and values (Gu, 2019). Existing has highlighted the pervasive influence of idol worship among contemporary undergraduates, with students from diverse academic disciplines and socioeconomic backgrounds demonstrating distinct patterns of idol preference (Peng and Sun, 2011).

However, with the development of the entertainment industry and the popularity of electronic products, college students' idol worship has become more common and fanatical than ever before, and the traditional “moral models” are gradually being replaced by “entertainment idols.” As the degree of idol worship deepens, individuals may even have pathological emotional involvement and behavioral manifestations (He and Sun, 2022). Excessive idol worship behaviors can lead to various negative emotions and behaviors among fans, damaging their studies, work and interpersonal relationships and even leading to dangerous behaviors such as self-injury or attempted suicide (Liu et al., 2022). Since 2020, the social media-based pop star idol worship subculture has brought about a significantly negative impact on the lives of fans and their families (Yin, 2020; Zhang and Negus, 2020). During emerging adulthood, college students confront the developmental task of reconstructing self-identity amid novel interpersonal and social relationships. Therefore, disentangling the determinants and mechanisms of idol worship holds significant implications for fostering healthy idol perception and promoting holistic development among university students.

### Maternal overprotection and idol worship

Negative parenting was strongly associated with idol worship (Cheung and Yue, 2012; Zhang and Li, 2006). Levy (1941) originally proposed the concept of parental overprotection. Overprotection is when parents are overly involved in their child's daily activities and experiences, limiting their child's autonomy. It is considered to be a negative parenting behavior (Jiang et al., 2010). Such behaviors may limit the child's ability to solve problems and interact with peers independently, resulting in an insecure parent-child attachment pattern (Affrunti and Woodruff-Borden, 2015; Knappe et al., 2012; Miller et al., 2018). The study has shown that idol worship is associated with insecure attachment (transfer of attachment partners) (Wang and Liu, 2010). According to attachment style theory, it has been suggested that the insecure attachment style that individuals establish with their parents in infancy may lead adolescents and adults to exhibit stalking, idols stalking and trying to get in close contact with their favorite idols (Kienlen, 1998). On this basis, McCutcheon et al. (2006) further hypothesized that insecure attachment idolatrous are more likely to have proximity behaviors than secure attachment-type idols. According to the self-determination theory, maternal overprotection will diminish children's sense of autonomy and competence, making it easier for them to seek approval and comfort through idol worship (Wang, 2017). After entering university, college students' ability to live and think independently has been continuously enhanced, and they have a certain sense of self (Shi and Fan, 2024). At this time, maternal overprotection will prevent college students from correctly understanding themselves (Jin and Li, 2023), making it easier for them to gain self-identity through idol worship. Accordingly, this study proposed Hypothesis 1: Maternal overprotection will significantly and positively predict college students' idol worship.

### The mediating role of reactive anger

Reactive anger refers to the frequency with which an individual produces angry emotions in response to a specific situational stimulus (Spielberger, 1988; Rippere, 1977; Yue et al., 2024; Jin et al., 2017a), which is an external manifestation of an individual's exposure to external circumstances, such as anger when an individual is criticized and mistreated (Luo et al., 2011). Bowlby (1969) argues that the core of secure attachment is that parents support children's self-directed exploration through “safe base” behavior, rather than excessive intervention. Overprotective parents can deprive their children of the opportunity to cope with challenges independently (Miller et al., 2015), leading to over-dependence on their mothers and developing insecure attachment styles (Favaretto et al., 2001). The parent-child attachment affects children's emotional regulation (Bowlby, 1969) and social skills (Boldt et al., 2014; Bartholomew and Horowitz, 1991). Previous research has indicated that individuals with insecure attachment may develop disorders of emotion regulation compared to securely attached individuals (Marganska et al., 2013; Goodall et al., 2012; Liu and Ma, 2019; Owens et al., 2018; Stroud et al., 2016). This process is supported by a neural mechanism: the prefrontal cortex of children with insecure attachment, which is responsible for emotion regulation, is inadequately activated (Warren et al., 2010). Thus, such children more likely to exhibit angry emotions (van der Voort et al., 2014; Mikulincer, 1998b; Troisi and D'Argenio, 2004). According to projection theory, individuals blame others for their socially unacceptable desires and behaviors, or manifest them in another form (Zhang, 2012). Within the context of idol fandom culture, the practice of idol worship becomes legitimized by their communities (Zhang and Negus, 2020), while anger—a socially “unacceptable” emotion—requires externalization through alternative forms. Idol worship thus serves as an ideal conduit for such emotional transmutation. Specifically, adolescents' idol worship usually reflects their long-suppressed subconscious thoughts and desires, which cannot be expressed directly but are realized through idol worship, the socially acceptable approach. The fast pace of modern life exacerbates the pressure of time compression, and college students tend to maximize their desires in a limited time (Zhang and Lv, 2010). Meanwhile, reactive anger may reflect attachment anxiety about overprotection and lack of autonomy, which leads adolescents to attempt to alleviate their anger by establishing a virtual emotional connection between the idol and themselves. Accordingly, this study proposed Hypothesis 2: Reactive anger may mediate the association between maternal overprotection and college students' idol worship.

### The mediating role of cybervictimization

Cybervictimization refers to an individual being repeatedly bullied by other individuals or groups in the process of using the internet, such as verbal intimidation and abuse, insults and malicious harassment (Jin et al., 2017b). A survey showed that nearly 70% of the youth interviewed said that they or those around them had experienced cyber violence and that young people were increasingly being attacked by online groups or individuals (Li, 2025). Some studies pointed out that overprotective parents could lead their children to adopt immature ways of solving problems when they encounter difficulties (He and Du, 2019), which would increase the likelihood of being victimized. According to social identity theory (Tajfel, 1978), individuals tend to define themselves by the group they belong to and behave similarly to the group. Thus, the individuals become aware of the emotion and value that being a member of the group brings to themselves. For victims of cyberbullying, idols and their fan groups may become an important source of social identity. Individuals will gain an identity in fan groups utterly different from their real lives, temporarily allowing them to put their real-life difficulties behind them (Cai and Ouyang, 2007). Additionally, victims of cyberbullying who feel isolated in real life are more inclined to seek idol worship as a means of emotional expression and social belonging. Accordingly, this study proposed Hypothesis 3: Cybervictimization may mediate the association between maternal overprotection and college students' idol worship.

### The chain mediating role of reactive anger and cybervictimization

Previous studies have shown that both reactive anger and cybervictimization are significantly associated with idol worship. However, the effect of reactive anger and cybervictimization on the association between maternal overprotection and college students' idol worship remains unclear. Therefore, this study explored the association between maternal overprotection and college students' idol worship by analyzing the chain mediation effect of the individual factor (reactive anger) and the environmental factor (cybervictimization).

The maternal overprotection may lead to the development of an insecure attachment type, which may first lead to the development of reactive anger in children (Zhang et al., 2014; Mikulincer, 1998a). According to attachment theory (Bowlby, 1969), overprotective parents can lead to insecure attachment (Xu et al., 2009; Chen and Zhang, 2011; Li et al., 2015). This attachment pattern tends to keep individuals in a state of emotional dysregulation (Crowell et al., 2020; Brumariu and Kerns, 2010). In certain situations, adolescents may develop reactive anger to express their resistance. This anger tendency as an individual factor could affect how children interact online, making them more likely to exhibit inappropriate social behavior and increasing the risk of being bullied online (Jin et al., 2017b; Sutton and Simons, 2021). Cybervictimization as an environmental factor further exacerbates individuals' dissatisfaction and isolation from the real world, prompting them to seek idol worship as a means of psychological compensation and social identity (Cai and Ouyang, 2007; Yue, 2004). Accordingly, this study proposed Hypothesis 4: Reactive anger and cybervictimization may play a chain mediating role between maternal overprotection and college students' idol worship.

**Figure 1:**
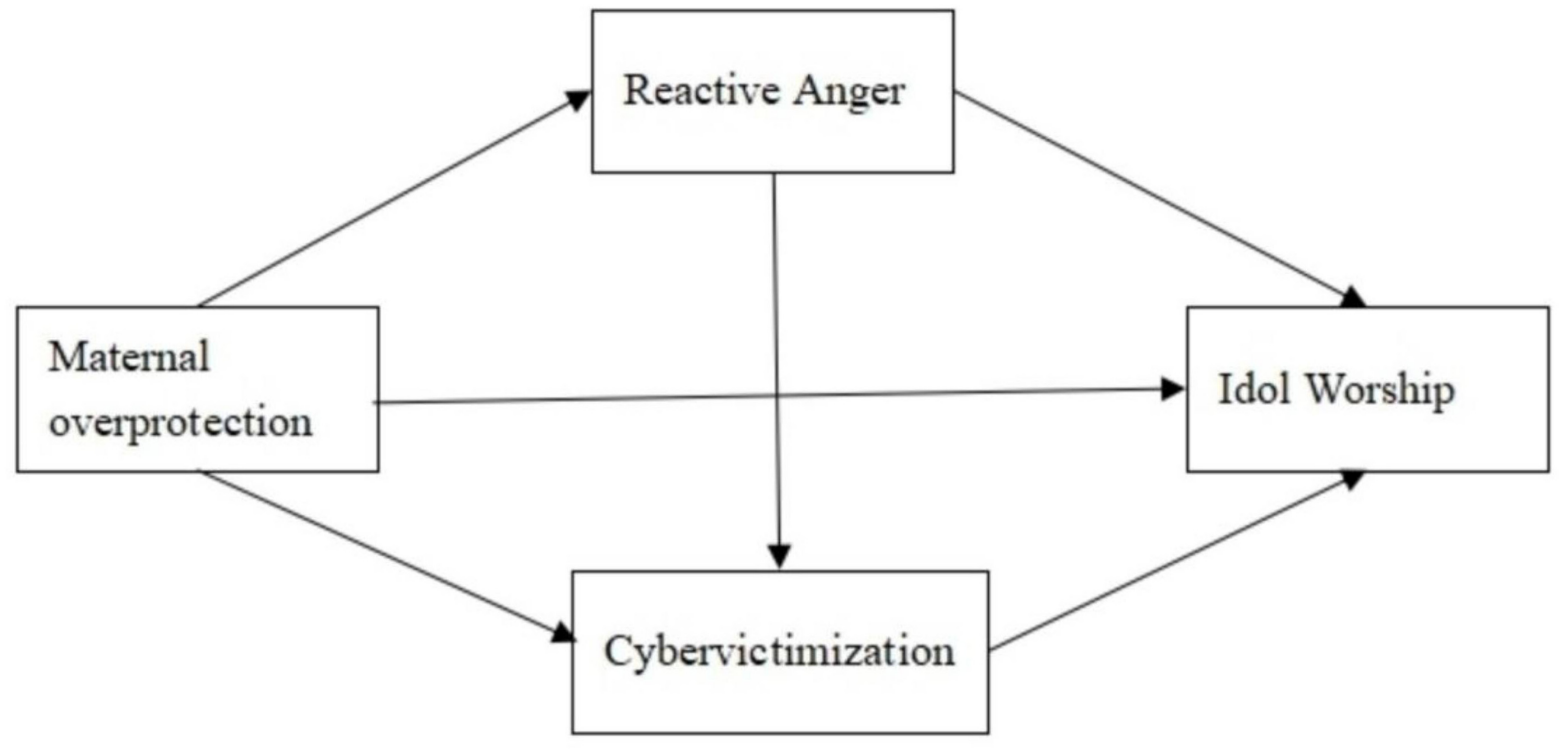
Path diagram of the influence of maternal overprotection on college students' idol worship.

In summary, this study constructs a theoretical analysis framework, as shown in Figure 1.

## Materials and methods

### Participants

The stratified cluster sampling method was used to conduct a questionnaire survey among college students at Weifang University of Science and Technology. A group test was conducted by class, and two classes were randomly selected from the four grades: freshman, sophomore, junior, and senior. A total of 905 questionnaires were distributed through the Questionnaire Star applet. The questionnaires were distributed from 7 to 12 January 2024. After removing invalid questionnaires such as regular answers, missing data and reverse answers, a total of 847 data were collected, with an effective recovery rate of 93.6%. The students' ages ranged from 17 to 24 years old (Mage = 19.57, SDage = 1.25). Approximately 76.50% of the students were female and 23.50% were male. Of the students, 64.10%, 17.00%, and 18.90% were from rural areas, suburban areas, and urban areas, respectively. In terms of academic year distribution, 31.80%, 50.50%, 12.60%, and 5.10% were freshmen, sophomores, juniors, and seniors, respectively. Notably, there is a large difference in the number of males and females because the participants are from humanities majors, but this is in line with the ratio of males and females in this major under Chinese culture. Moreover, seniors were underrepresented, likely due to their lower presence on campus.

### Procedures

According to the declaration of Helsinki, this study design had passed the ethical review of the Human Research Ethics Committee of Weifang University of Science and Technology. Participants participated voluntarily, were informed of the purpose of the survey, answered methods and precautions, and signed informed consent forms. Before the test, the questionnaire data collectors were uniformly trained in the testing process, key points, and methods. In the test, mature scales were used for online measurement, questionnaires were sent to students through WeChat, and students completed the questionnaire independently on computers or mobile phones, emphasizing the principles of voluntary filling, the confidentiality of information, and anonymous filling.

### Variables and measures

#### Maternal overprotection

Maternal overprotection was measured using the Short-Egna Minnen av Barndoms Uppfostran-Chinese version (S-EMBU-C) developed by Arrindell et al. (2001) and revised by Jiang et al. (2010), which was reliable among Chinese college students (Cronbach's alpha = 0.78). This full scale consists of 42 items divided into two sections for mothers and fathers and three dimensions. Based on the research purpose, we only used the “maternal overprotection” dimension of the mother's subscale. It includes 8 items (for example, “I think my mother's fears about what might happen to me were exaggerated and excessive”). A 4-point scale was used ranging from 1 (never) to 4 (always). Higher average scores indicated higher levels of maternal overprotection. The Cronbach's alpha for this scale was 0.77.

#### Idol worship

The Celebrity Attitude Scale (CAS-R) developed by McCutcheon et al. (2002) and revised by Peng et al. (2010) was used to assess the degree of idol worship, which was reliable among Chinese college students (Cronbach's alpha = 0.88). It includes 27 items (for example, “I pay special attention to the details of the life of my favorite idols”). A 5-point Likert scale was used, ranging from 1 (strongly disagree) to 5 (strongly agree), with higher average scores indicating higher levels of idol worship involvement. The Cronbach's alpha for this scale was 0.97.

#### Reactive anger

Reactive anger was measured using the subscale Trait Anger Scale (TAS) developed by Spielberger (1988) and translated and revised by Luo et al. (2011), which was reliable among Chinese college students (Cronbach's alpha = 0.80). This scale consists of 10 items divided into two dimensions. Based on the research purpose, we only used the “reactive anger” dimension, which included six items (for example, “I get angry when someone else makes a mistake and delays my progress”). A 4-point scale was used, ranging from 1 (hardly) to 4 (always), with higher average scores implying a higher level of reactive anger. The Cronbach's alpha for this scale was 0.86.

#### Cybervictimization

Cybervictimization was measured using the scale revised by Hood and Duffy (2018) and translated by Jin (2021), which was reliable among Chinese college students (Cronbach's alpha = 0.87). The questionnaire has fewer questions and focuses on the assessment of bullying and being bullied on social networking platforms. The questionnaire was translated back into Chinese and English and cultural adaptation was carried out. This scale consists of seven items (for example, “Someone has laughed or teased me on social networks”), using a 5-point Likert-type scale ranging from 1 (never) to 5 (always). Participants evaluated their experiences of being bullied on each item. Higher scores indicated higher levels of cybervictimization. The Cronbach's alpha for this scale was 0.96.

### Data analysis

SPSS 26. 0 was used to conduct the statistical analysis. First, Harman's single-factor test was used to test for the common method bias (Aguirre-Urreta and Hu, 2019). According to principal component analysis, there were six factors with eigenvalues >1. The first factor explained a variation of 35.564%, less than the critical standard of 40% (Deng et al., 2018), suggesting that there was no significant common method bias in this study. Then, participants' characteristics were analyzed descriptively, including means and standard deviations. Pearson correlation analyses examined the relationships among all variables. Finally, Hayes' PROCESS macro (Model 6) was used for mediation analysis (Hayes, 2013). The data was bootstrapped with 5,000 samples to obtain 95% confidence intervals (CI) (Shrout and Bolger, 2002). The mediating effect was considered significant if the upper and lower values of the 95% confidence interval did not contain 0 between them. Among them, the independent variable was maternal overprotection, the mediating variables were reactive anger (mediating variable 1) and cybervictimization (mediating variable 2), the dependent variable was idol worship (Y). Gender was significantly associated with maternal overprotection, cybervictimization and idol worship, and this study used gender as a covariate in subsequent analyses. However, there was no significant correlation between other demographic variables and the study variables, so they were not considered as control variables in this study.

## Results

### Descriptive statistical analysis

The descriptive statistics and results of the correlation analysis of each variable are presented in Table 1. The findings indicate that maternal overprotection was significantly and positively associated with reactive anger (*r* = 0.349, *p* < 0.001), cybervictimization (*r* = 0.272, *p* < 0.001) and idol worship (*r* = 0.113, *p* < 0.01). Reactive anger was significantly and positively associated with cybervictimization (*r* = 0.465, *p* < 0.001) and idol worship (*r* = 0.197, *p* < 0.001). Cybervictimization was significantly and positively associated with idol worship (*r* = 0.319, *p* < 0.001).

**Table 1:** Descriptive statistics and correlations of study variables.

**Variable**	**M ±SD**	**1**	**2**	**3**	**Skewness (*SE*)**	**Kurtosis (*SE*)**
1. Maternal overprotection	1.97 ± 0.49				0.71 (0.08)	0.81 (0.17)
2. Reactive anger	1.57 ± 0.57	0.349^***^			1.38 (0.08)	2.71 (0.17)
3. Cybervictimization	1.30 ± 0.68	0.272^***^	0.465^***^		2.86 (0.08)	9.01 (0.17)
4. Idol worship	2.31 ± 0.91	0.113^**^	0.197^***^	0.319^***^	0.43 (0.08)	−0.12 (0.17)

** *p* < 0.01,

*** *p* < 0.001.

### Mediation analysis

To ensure that there were clear boundaries between the variables and that the model was not distorted during regression analysis, we further measured the variance expansion factor (VIF) between the variables. The results of regression analysis (see Table 2) showed that the VIF values between maternal overprotection (independent variable), reactive anger (mediating variable 1), cybervictimization (mediating variable 2), and idol worship (dependent variable) are all smaller than 5 (1.15 < VIF < 1.40), so there is no serious multicollinearity problem between the variables.

**Table 2:** Linear regression analysis coefficient^a^ results.

**Predictors**	**Unstandardized coefficients**		**Standardized coefficients**	***t***	**Sig**.	**Collinearity statistics**	**VIF**
	***B***	**Std. error**	**Beta**			**Tolerance**	
Constant	1.607	0.131		12.290	0		
Cybervictimization	0.388	0.050	0.288	7.761	0	0.770	1.299
Reactive anger	0.094	0.062	0.058	1.524	0.128	0.730	1.369
Maternal overprotection	0.026	0.065	0.014	0.403	0.687	0.863	1.159

a Dependent variable: idol worship.

**Figure 2:**
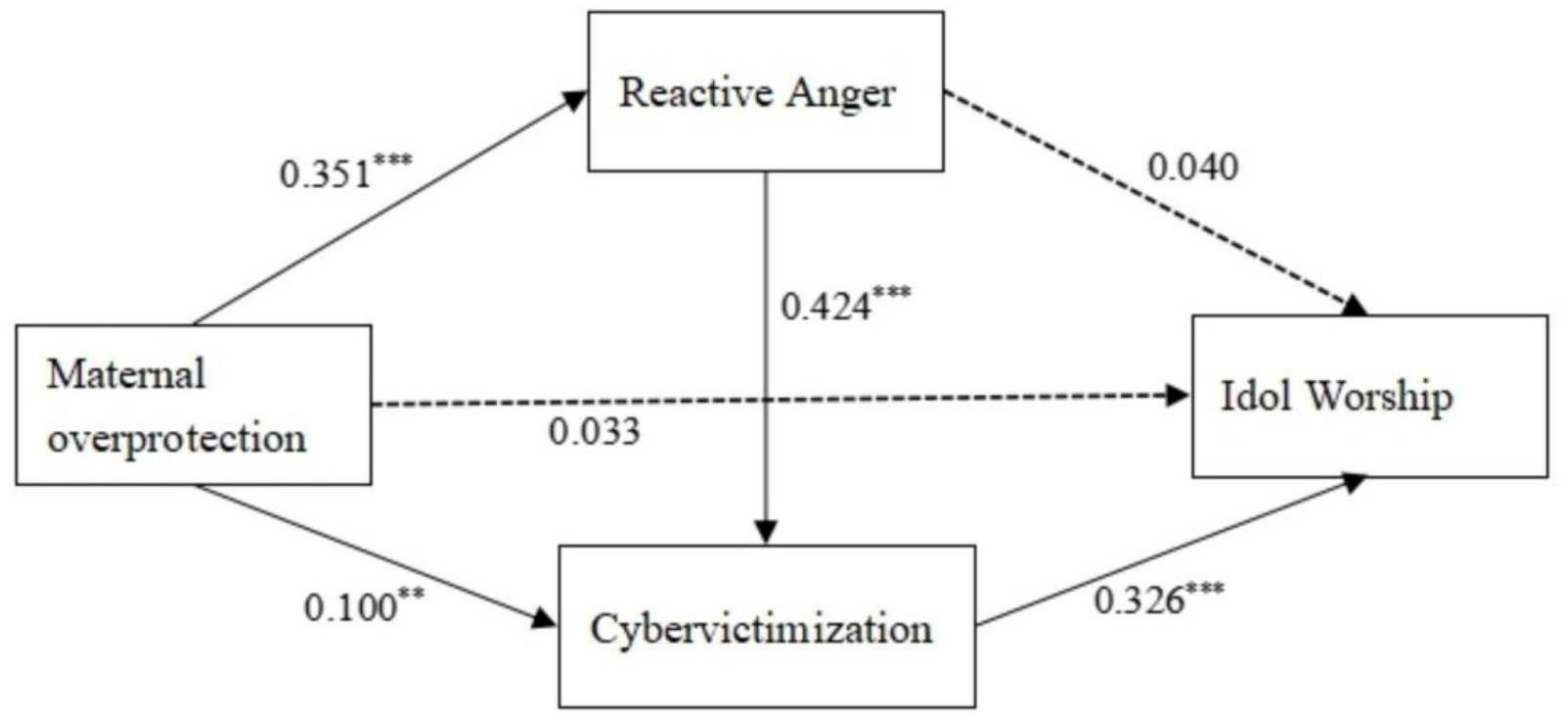
The final mediating effects diagram the influence of maternal overprotection on college students' idol worship. **p < 0.01, ***p < 0.001.

The results of the regression analysis are summarized in Table 3, Figure 2. The results showed that after controlling for the effect of gender, maternal overprotection significantly and positively predicted idol worship (β = 0.128, *t* = 3.726, *p* < 0.001), reactive anger (β = 0.351, *t* = 10.772, *p* < 0.001) and cybervictimization (β = 0.100, *t* = 3.138, *p* < 0.001). Reactive anger significantly and positively predicted cybervictimization (β = 0.424, *t* = 13.412, *p* < 0.001). When maternal overprotection, reactive anger, and cybervictimization entered the regression equation at the same time, maternal overprotection (β =0.033, *t* = 0.943, *p* > 0.05) and reactive anger (β = 0.040, *t* =1.062, *p* > 0.05) were not a direct predictor of idol worship, and cybervictimization significantly and positively predicted idol worship (β =0.326, *t* = 8.714, *p* < 0.001).

**Table 3:** Regression analysis of the relationship between the variables (*n* = 847).

**Regression equation** **Independent variable**	**Dependent variable**	**Overall fit index**			**Significance of regression coefficient**	
		***R***	***R** ^2^*	***F***	β	***t***
Idol worship	Gender	0.157	0.025	10.634	0.110	3.201^**^
	Maternal overprotection				0.128	3.726^***^
Reactive anger	Gender	0.349	0.122	58.633	0.012	0.368
	Maternal overprotection				0.351	10.772^***^
Cybervictimization	Gender	0.511	0.261	99.256	−0.178	−5.946^***^
	Maternal overprotection				0.100	3.138^**^
	Reactive anger				0.424	13.412^***^
Idol worship	Gender	0.362	0.131	31.685	0.166	5.002^***^
	Maternal overprotection				0.033	0.943
	Reactive anger				0.040	1.062
	Cybervictimization				0.326	8.714^***^

** *p* < 0.01,

*** *p* < 0.001.

Next, the Bootstrap method was used to further analyse the chain mediation effects of reactive anger and cybervictimization on the association between maternal overprotection and college students' idol worship. The mediation analysis (see Table 4) revealed that the total mediation effect value was 0.176, accounting for 74.26% of the total effect of maternal overprotection and college students' idol worship (effect value 0.237). Three pathways constituted this mediating effect: first, maternal overprotection → reactive anger → idol worship, with an effect value of 0.026 (10.97%) and 95% confidence interval containing 0, indicating that the mediating effect of reactive anger is not significant. Second, maternal overprotection → cybervictimization → idol worship, with an effect value of 0.060 (25.32%) and 95% confidence interval for [0.022, 0.105], indicated that the mediating role of cybervictimization is significant. Hence, Hypothesis 3 was supported. Third, maternal overprotection → reactive anger → cybervictimization → idol worship, with an effect value of 0.090 (37.97%) and 95% confidence interval for [0.050, 0.140], indicated that the chain mediation that maternal overprotection increased levels of reactive anger and cybervictimization, and then increases the level of idol worship of college students. Hence, Hypothesis 4 was supported. In addition, we compared the effect sizes of the three profile paths. The results showed that there were no significant differences between the three indirect pathways (see Table 4).

**Table 4:** The direct and indirect effect of the chain mediation model.

	**Effect value**	**Boot SE**	**Bootstrap 95%CI**		**Proportion of relative effect**
			**Boot LLCI**	**Boot ULCI**	
Direct effect	0.061	0.065	−0.066	0.187	25.74%
X → M1 → Y	0.026	0.028	−0.028	0.084	10.97%
X → M2 → Y	0.060	0.021	0.022	0.105	25.32%
X → M1 → M2 → Y	0.090	0.023	0.050	0.140	37.97%
Total indirect effect	0.176	0.044	0.096	0.268	74.26%
Total effect	0.237	0.064	0.112	0.362	
C1			−0.11	0.04	
C2			−0.14	0.01	
C3			−0.09	0.02	

X for maternal overprotection, Y for idol worship, M1 for reactive anger and M2 for cybervictimization. C1=X → M1 → Y minus X → M2 → Y; C2=X → M1 → Y minus X → M1 → M2 → Y; C3=X → M2 → Y minus X → M1 → M2 → Y.

Based on the cross-sectional data used in this study, in order to further explore the causal relationship between the variables, we took idol worship as the independent variable, maternal overprotection as the dependent variable, and reactive anger and cybervictimization as the two mediating variables, but the chain mediating effect was not established.

## Discussion

The study found that maternal overprotection significantly and positively predicted idol worship. Studies have proved that parenting styles are an essential cause of idol worship in individuals (Qiao et al., 2023). Maternal overprotection may negatively affect adolescents' self-identity (Cui, 2021; Tang and Li, 2022) and self-confidence (Zhu and Liu, 2003). According to the theory of self-determination, autonomy has an important impact on individual mental health (Ryan et al., 2016; Vansteenkiste et al., 2006). However, maternal overprotection may limit their children's autonomy and independence. This behavior of not giving children ample opportunities to explore can weaken their ability to establish self-identity, which can lead to confusion and anxiety. Under this circumstance, seeking external approval becomes a relatively viable option for adolescents. They crave recognition and affirmation from others because they are unable to determine their worth from within. Therefore, idol worship can be seen as a compensatory mechanism. By imitating the behavior and values of their idols, adolescents try to find their place in society and gain recognition from others. They hope to boost their self-esteem and sense of identity by establishing a connection with their idols, because their idols' success can be seen as their own. In collectivist cultures, family relationships have a more profound impact on individual behavior, potentially amplifying the association between maternal overprotection and idol worship. For example, China's concept of “filial piety” may force adolescents to suppress direct resistance to parental control and instead express their need for autonomy indirectly through idol worship. Conversely, in individualistic cultures, adolescents are more likely to cope with overprotection through independent decision-making (such as moving out of their family) rather than idol worship. The impact of maternal overprotection on college students' idol worship is a critical issue. This result implied that maternal overprotection may hinder college students' development of autonomous identity, potentially driving them to seek compensatory emotional anchors and idealized models through idol worship. By studying the mechanism of its influence in-depth and making targeted suggestions, college students can grow up healthy and reduce the behavior of over-worship of idols.

In this study, the mediating role of reactive anger was not established and inconsistent with the expected Hypothesis 2. From the perspective of attachment theory, overprotective parenting often leads to insecure attachment patterns (Favaretto et al., 2001), which may directly drive individuals to seek alternative emotional connections (Hazan and Shaver, 1987). Specifically, the insecurity of insecure attachment may be transformed into the emotional projection of the idealized image of idols, and this process may bypass the mediation of specific emotions and directly form a compensation mechanism for idol worship. Self-determination theory provides a new dimension for understanding this phenomenon. Maternal overprotection can lead to adolescents' anxiety (Breinholst et al., 2019) and social withdrawal (Van Petegem et al., 2022; Liu et al., 2023), thereby limiting adolescent autonomy development (Deci and Ryan, 2000). When an idol succeeds as a mirror image of the ideal self, the individual will have a great sense of psychological satisfaction and accomplishment (Zhang and Zuo, 2006), and, to some extent, achieve the satisfaction of autonomy. However, this compensatory mechanism relies more on the individual's long-term psychological need for autonomy than on immediate emotional responses (Gagné and Deci, 2005). It is necessary to note that the reactive anger measured in this study is a state-like emotion indicator, which may lead to the failure of the mediating effect. In addition, in the context of collectivist culture, individuals may perceive maternal overprotection as caring rather than controlling, and this cognitive bias may weaken the generation of anger.

The study found that maternal overprotection influenced idol worship through the mediating role of cybervictimization, in line with the expected Hypothesis 3. According to attachment theory, the early parent-child relationship and the level of closeness between mother and child significantly impact an individual's psychological development and social relationship formation. From the perspective of attachment theory, overprotection is essentially intrusive parenting (Barber, 2002), which makes adolescents overdependent on their mothers and forms insecure attachment patterns. This pattern leads lead to a lack of proper understanding, coping, and self-protection skills in the cyber world among college students (Valdez, 2016). making them more likely to be victims. According to the self-determination theory, long-term excessive intervention may lead to a lack of self-confidence (Claes et al., 2015; Zhu and Liu, 2003) and self-esteem (Yin, 2004) due to a lack of autonomy, making them more sensitive to attacks from the Internet, thus making the impact of bullying more significant. Meanwhile, maternal overprotection may lead to social isolation of college students from their peers (Xu and Yan, 2023), and individuals who lack social support networks are more likely to become targets of cyberbullying. In this context, idol worship becomes an essential way for them to seek comfort and support their feelings. By imitating and following their idols, they try to compensate for their lack of self-identity, as well as to escape the negative experiences they suffer in real life. This finding highlighted the critical need for anti-cyberbullying interventions in schools and communities, as reducing adolescents' cybervictimization may effectively disrupt the pathway from maternal overprotection to excessive idol worship. Therefore, improving maternal parenting style can reduce the situation of college students suffering from cyberbullying, which can effectively reduce the level of idol worship.

This study also found that maternal overprotection had an effect on idol worship through the chain mediation of reactive anger and cybervictimization, which verified Hypothesis 4. According to self-determination theory, the basis of good individual development is the fulfillment of autonomy needs (Deci and Ryan, 2000), while maternal overprotection can hinder or inhibit the development of an individual's autonomy needs (Van Petegem et al., 2020), depriving children of the opportunity to make and act on their own decisions (Pu and Zhang, 1996), thus making them feel depressive (Xu, 2018). In response, adolescents may display reactive anger. This angry emotion is a response to the maternal overprotective behavior and a way for the child to try to regain a sense of autonomy and control. Moreover, the anonymity and disinhibition effects of cyberspace (Zhou and Liu, 2016) may reinforce the intensity of reactive anger. This phenomenon amplified emotional state can lead to individuals being more likely to overreact to negative remarks or aggressive behavior online, making individuals the target of cyberbullying. The study has been conducted to prove that trait anger promotes hostile attribution bias (Li and Xia, 2021). Individuals with high levels of reactive anger are more likely to pick up hostile cues from the incident, interpret the ambiguous behavior of others as malicious, and attack others, which is likely to provoke a stronger group pushback. The experience of being bullied often has a negative impact on an individual's mental health (Bannink et al., 2014; Patchin and Hinduja, 2011) and self-esteem (Didden et al., 2009; Strøm et al., 2014). In this context, idol worship can be seen as a coping mechanism to help individuals restore self-esteem and achieve self-identity and emotional attachment after experiencing cyberbullying. This finding underscored the necessity of integrating anger management training into youth mental health programs, as teaching adolescents adaptive emotion-regulation strategies can disrupt the spillover of reactive anger into online conflicts, thereby reducing dual risks of cybervictimization and compensatory idolization.

## Implications

### Theoretical implications

Based on self-determination theory, this study suggests that maternal overprotection positively promotes idol worship, which validates and expands the application of self-determination theory to parenting and contributes to an in-depth understanding of the topic between adolescent idol worship and parenting. In addition, this study validated the cascading mediating effects of reactive anger and cybervictimization between maternal overprotection and idol worship. For the first time, reactive anger was incorporated into a self-deterministic framework, revealing that autonomy need deprivation triggers emotional responses that form a behavioral chain through hostile attributional bias, and refining the behavioral mechanism of self-recovery after an individual experiences cyberbullying. It is also worth noting that this study suggests that the “filial piety” ethic in collectivist cultures reinforces the association between maternal overprotection and idol worship, providing new evidence for the cross-cultural applicability of self-determination theory. Second, this study validates the mediating role of cybervictimization between maternal overprotection and idol worship, expanding attachment theory in a new dimension. Extending the study of attachment theory from real-life relationships to cyberspace, reveals how maternal overprotection works in conjunction with online risk to affect adolescent mental health. Finally, this study combines self-determination theory and attachment theory to propose a mediation model of interlocking, which involves psychological compensation for individuals' blocked intrinsic needs but also covers external risks caused by interpersonal relationship deficits, providing a more comprehensive theoretical support for understanding adolescent developmental problems with more comprehensive contents and broader perspectives.

### Practical implications

The mechanism of maternal overprotection on adolescent idol worship reflects the important influence of family upbringing style on individual psychological development. This influence is of great significance in guiding the practice of family education and improving the psychological health of adolescents. Specifically, measures can be taken in the following three ways:

#### Family improvements: stepped exposure interventions

First, promoting parent-child communication and emotional support is crucial. Regular communication, listening to children's thoughts, and showing love help build trust and emotional bonds. Second, parents should establish healthy parent-child boundaries and respect their children's personal space and privacy to foster their independence and autonomy. Finally, parents can use the overprotective subscale in the localized Parenting Styles Scale (Yue et al., 1993) to identify overprotective behaviors like substitute decision-making. Self-intervention based on stepped exposure therapy (Chorpita et al., 2011) can be applied. For example, start by letting children organize study time, then gradually extends to social and vocational choices.

#### Emotion regulation training: cognitive—physiological—environmental support

Schools and social organizations can offer emotion management training, focusing on cognitive, physiological, and environmental aspects. This helps students understand and regulate emotions, and develop self- and emotional awareness.

First, school mental health programmes should introduce emotional granularity training (Kashdan et al., 2015). Students are taught to use precise words like “frustration” and “aggression” instead of general anger expressions. They also learn to distinguish between “hostile attributions” (e.g., “he deliberately humiliated me”) and “non-malicious attributions” (e.g., “he may not have understood the context”), effectively guiding adolescents to identify and manage emotions cognitively. Secondly, regarding physiological conditioning, schools can introduce the 8-week positive thinking programme designed by Tang et al. (2015) to focus on training students in “body scanning” and “awareness-reactivity” techniques. When triggered by anger, students learn to notice physical signs like clenched fists and shortness of breath without reacting. Meta-analyses have shown this boosts emotion regulation by 0.61 standard deviations (Goldberg et al., 2018). Finally, schools can hold “co-regulation of emotions” workshops. These help parents learn to respond properly to children's emotions. For instance, when kids are angry, parents should avoid negative or overprotective responses and use empathetic statements instead.

#### Cybervictimization prevention: platform-school joint prevention mechanism

Schools and families must boost youth cybersecurity education, covering proper net use, privacy safeguarding, and cyberbullying prevention. The Internet Information Office can create a cross-platform reporting system. Social platforms such as TikTok and Weibo should notify schools of major student-related cyberbullying within 24 h, building a “monitor-warn-handle-feedback” loop. Additionally, schools and social groups can provide psychological aid to cybervictimization youth. For example, school counseling centers can offer a 12-week cognitive processing therapy CPT (Resick et al., 2017), where victimization experiences are transformed into “survivor stories” through writing exercises (like “I was attacked, but supported by my friends”). Schools can also recruit students who have successfully navigated cyberbullying to form peer support communities to share strategies for coping with cyberbullying through regular salons.

## Limitations and future research

Although this study conducted a preliminary exploration of the relationship between maternal overprotection, reactive anger, cybervictimization, and idol worship, there are still some limitations that need to be further improved and addressed in future research. First, in terms of the sample selection, the sample of this study is relatively homogeneous. The subjects in this study were a group of college students, ignoring the critical developmental stage of early adolescence (12–15 years old). Individuals at this stage are in a period of exploding need for autonomy and have the most intense conflict with mother's control. Idol worship behaviors tend to be more impulsive and emotionally attached. The response patterns of college students, who are already partially independent decision-makers, may weaken the actual intensity of the impact of overprotective behaviors. Future studies may consider expanding the sample to cover more age groups to verify the robustness and reliability of the findings. Second, future research can further test the universality of the theoretical framework by expanding the boundaries of disciplines. For example, we select fields significantly different from the current disciplines (for example, gender-balanced majors in humanities and social sciences or gender-unbalanced majors in science and engineering) to systematically investigate the moderating effect of group composition variables on research results. It is also important to note that the non-normal distribution of cybervictimization data may pose a challenge to statistical model assumptions. However, through statistical processing, the results of the study were proved to be reliable. Future studies can be further validated using larger samples or latent variable models. Third, in terms of measurement methodology, the risk of common methodological biases associated with self-reporting has not been effectively controlled. In addition, this study reduces the characteristic of “maternal overprotection” to a static trait and ignores its dynamic evolution, which is one-sided. In reality, family parenting styles may fluctuate in response to situational factors such as children's age and stressful school events, and this dynamic is difficult to capture in a cross-sectional design. Future studies could adopt a longitudinal tracking design to follow the developmental changes of the research participants, such as capturing the fluctuation patterns of mother's controlling behaviors and idol worship intensity through dynamic monitoring, with special attention to the conditions of mechanism activation after stressful events such as exam weeks and family conflicts to gain a deeper understanding of the causal relationships between maternal overprotection, reactive anger, cybervictimization, and idol worship. Finally, China's fertility policy has shifted from encouraging family planning to a two-child policy, which means that family outcomes in China are changing or have changed. Unlike in the past when there were mostly only children, more and more families now have at least two children. Hu et al. (2018) found that in one-child families, parents adopt parenting styles that aim to make their children independent. However, we do not know whether the parenting styles adopted by Chinese families will change as the family structure shifts. This also deserves to be the focus of future research.

## Data Availability

The raw data supporting the conclusions of this article will be made available by the authors, without undue reservation.

## References

[B1] Affrunti N. W. Woodruff-Borden J. (2015). Parental perfectionism and overcontrol: examining mechanisms in the development of child anxiety. J. Abnorm. 43, 517–529. 10.1007/s10802-014-9914-5 25030793

[B2] Aguirre-Urreta M. Hu J. (2019). Detecting common method bias: performance of the harman's single-factor test. Data. Base. Adv. Inf. Syst. 50, 45–70. 10.1145/3330472.3330477

[B3] Arrindell W. Richter J. Eisemann M. Gärling T. Rydén O. Hansson S.. (2001). The short-EMBU in East-Germany and Sweden: a cross-national factorial validity extension. Scand. J. Psychol. 42, 157–160. 10.1111/1467-9450.00226 11321639

[B4] Bannink R. Broeren S. van de Looij-Jansen P. M. de Waart F. G. Raat H. (2014). Cyber and traditional bullying victimization as a risk factor for mental health problems and suicidal ideation in adolescents. PLoS ONE 9:e94026. 10.1371/journal.pone.0094026 24718563 PMC3981739

[B5] Barber B. K. (Ed.). (2002). Intrusive Parenting: How Psychological Control Affects Children and Adolescents. American Psychological Association.

[B6] Bartholomew K. Horowitz L. M. (1991). Attachment styles among young adults: a test of a four-category model. J. Pers. Soc. Psychol. 61, 226–244. 10.1037/0022-3514.61.2.226 1920064

[B7] Boldt L. J. Kochanska G. Yoon J. E. Koenig Nordling J. (2014). Children's attachment to both parents from toddler age to middle childhood: links to adaptive and maladaptive outcomes. Attach. Hum. Dev. 16, 211–229. 10.1080/14616734.2014.889181 24605850 PMC3997589

[B8] Bowlby J. (1969). Attachment and Loss: Vol. 1. Attachment. New York: Basic Books.

[B9] Breinholst S. Tolstrup M. Esbjørn B. H. (2019). The direct and indirect effect of attachment insecurity and negative parental behavior on anxiety in clinically anxious children: it's down to dad. Child Adolesc. Ment. Health 24, 44–50. 10.1111/camh.12269 32677229

[B10] Brumariu L. E. Kerns K. A. (2010). Parent-child attachment and internalizing symptoms in childhood and adolescence: a review of empirical findings and future directions. Dev. Psychopathol. 22, 177–203. 10.1017/S0954579409990344 20102655

[B11] Cai Q. Ouyang J. (2007). Fans' culture in the vision of society and communication. J. Jiangsu Ocean Univ. 5, 73–76. Available online at: https://kns.cnki.net/nzkhtml/xmlRead/xml.html?pageType=web&fileName=HHGX200702019&tableName=CJFDTOTAL&dbCode=CJFD&fileSourceType=1&appId=KNS_BASIC_PSMC&invoice=V6xx9NKMMOcgbuaEy9JX63cTqUtOgPXaA3uw6yQnZzcKHV2kBBUW29MQVSdUJF7Fy73tAK5PB/Y5bROtjgwo6tGxbicxywZoG30kSSzH9tgKJEoF672fN8Cm2MDv0hzTSYo+6vYlDqRT8kWiaQgkZwsoGxoPATWsf3sV9uGLgag=

[B12] Chen X. Q. Zhang J. F. (2011). Relationship between normal college students' adult attachment and parenting style. Chin. J. Health Psychol. 19, 1003–1005. 10.13342/j.cnki.cjhp.2011.08.027

[B13] Cheung C. K. Yue X. D. (2012). Idol worship as compensation for parental absence. Int. J. Adolesc. Youth 17, 35–46. 10.1080/02673843.2011.649399

[B14] Chorpita B. F. Daleiden E. L. Ebesutani C. Young J. Becker K. D. Nakamura B. J.. (2011). Evidence-based treatments for children and adolescents: an updated review of indicators of efficacy and effectiveness. Clin. Psychol. Sci. Pract. 18, 154–172. 10.1111/j.1468-2850.2011.01247.x

[B15] Claes L. Bouman W. P. Witcomb G. Thurston M. Fernandez-Aranda F. Arcelus J. (2015). Non-suicidal self-injury in trans people: associations with psychological symptoms, victimization, interpersonal functioning, and perceived social support. J. Sex. Med. 12, 168–179. 10.1111/jsm.12711 25283073

[B16] Crowell S. E. Vlisides-Henry R. D. Kaliush P. R. (2020). “Emotion generation, regulation, and dysregulation as multilevel transdiagnostic constructs,” in The Oxford Handbook of Emotion Dysregulation, eds T. P. Beauchaine and S. E. Crowell (Oxford University Press), 85–97.

[B17] Cui P. (2021). The influence of family parenting style on college students' sense of collective responsibility: the mediating role of self-identity. Cult. Ind. 14, 120–121. Available online at: https://kns.cnki.net/nzkhtml/xmlRead/xml.html?pageType=web&fileName=WHCC202107045&tableName=CJFDTOTAL&dbCode=CJFD&fileSourceType=1&appId=KNS_BASIC_PSMC&invoice=UEopMIO/y9cgtCoHa+psBI1ax4waK7MFsQcutRFX2Eg8s8UOQA/yLGvRXCT3C+wIbXZ7UHS08etZ3ulfOtIVg/6YhkQlTCwm0/yEFMQoLBM6Wy1SqklzPtUDA7meogCkRxIMDz77d/H+h9qpJedLC7yEDX+cCf7MS3ADLCyDQxM=

[B18] Deci E. L. Ryan R. M. (2000). The “what” and “why” of goal pursuits: human needs and the self-determination of behavior. Psychol. Inq. 11, 227–268. 10.1207/S15327965PLI1104_01

[B19] Deng W. G. Li X. Y. Chen B. Luo K. Zeng X. Y. (2018). Analysis on application of common methods bias test to psychological studies during recent five years in china. J. Jiangxi Norm. Univ. Nat. Sci. 42, 447–453. 10.16357/j.cnki.issn1000-5862.2018.05.02

[B20] Didden R. Scholte R. H. Korzilius H. De Moor J. M. Vermeulen A. O'Reilly M.. (2009). Cyberbullying among students with intellectual and developmental disability in special education settings. Dev. Neurorehabil. 12, 146–151. 10.1080/17518420902971356 19466622

[B21] Favaretto E. Torresani S. Zimmermann C. (2001). Further results on the reliability of the Parental Bonding Instrument (PBI) in an Italian sample of schizophrenic patients and their parents. J. Clin. Psychol. 57, 119–129. 10.1002/1097-4679(200101)57:1<119::aid-jclp12>3.0.co;2-2 11211280

[B22] Gagné M. Deci E. L. (2005). Self-determination theory and work motivation. J. Organ. Behav. 26, 331–362. 10.1002/job.322

[B23] Goldberg S. B. Tucker R. P. Greene P. A. Davidson R. J. Wampold B. E. Kearney D. J.. (2018). Mindfulness-based interventions for psychiatric disorders: a systematic review and meta-analysis. Clin. Psychol. Rev. 59, 52–60. 10.1016/j.cpr.2017.10.011 29126747 PMC5741505

[B24] Goodall K. Trejnowska A. Darling S. (2012). The relationship between dispositional mindfulness, attachment security and emotion regulation. Pers. Indiv. Differ. 52, 622–626. 10.1016/j.paid.2011.12.008

[B25] Gu H. K. (2019). “Research on the present situation of contemporary college students' Idol worship and countermeasures,” in 2019 3rd International Conference on Economic Development and Education Management (ICEDEM 2019) (Atlantis Press), 41–43.

[B26] Hayes A. F. (2013). Introduction to Mediation, Moderation, and Conditional Process Analysis: A Regression-Based Approach. NewYork: The Guilford Press.

[B27] Hazan C. Shaver P. (1987). Romantic love conceptualized as an attachment process. J. Pers. Soc. Psychol. 52, 511–524. 10.1037/0022-3514.52.3.511 3572722

[B28] He S. Du B. L. (2019). Psychological resilience of vocational students in ethnic areas: the influence of coping style and parental rearing style. Chin. J. Health Psychol. 27, 1266–1270. 10.13342/j.cnki.cjhp.2019.08.039

[B29] He Y. Sun Y. (2022). Breaking up with my idol: a qualitative study of the psychological adaptation process of renouncing fanship. Front. Psychol. 13:1030470. 10.3389/fpsyg.2022.1030470 36591090 PMC9803266

[B30] Hood M. Duffy A. L. (2018). Understanding the relationship between cyber-victimisation and cyber-bullying on social network sites: the role of moderating factors. Pers. Indiv. Differ. 133, 103–108. 10.1016/j.paid.2017.04.004

[B31] Hu R. Ma Z. Xue Z. Tang Y. Wang L. Wang Z. (2018). Effects of child gender and parenting style on neurobehavior. Chin. J. Sch. Health 39, 136–138. 10.16835/j.cnki.1000-9817.2018.01.044

[B32] Jiang J. Lu Z. R. Jiang B. J. Xu Y. (2010). Revision of the short fom Egna M innen av Barndom sUppfostran for Chinese. Psy. Dev. Edu. 26, 94–99. 10.16187/j.cnki.issn1001-4918.2010.01.017

[B33] Jin T. L. Lu G. Z. Zhang L. Jin X. Z. Wang X. Y. (2017a). The effect of trait anger on online aggressive behavior of college students: the role of moral disengagement. Psychol. Dev. Educ. 33, 605–613. 10.16187/j.cnki.issn1001-4918.2017.05.11

[B34] Jin T. L. Lu G. Z. Zhang L. Wei L. C. Ma X. Suo H. X. (2017b). Psychological maltreatment and cyber victimizationin adolescents: mediating of social anxiety. Chin. J. Clin. Psychol. 25, 167–170. 10.16128/j.cnki.1005-3611.2017.01.037

[B35] Jin X. Y. (2021). The relationship between college students' cyberbullying and cyberbullying: a moderated chain mediation model (Unpublished mater's thesis). Tianjin Normal University, Xiqing, China.

[B36] Jin Y. D. Li X. S. (2023). The effect of parenting style on time attitude of college students: the mediating role of core self-evaluation. Psychol. Explor. 43, 472–480. Available online at: https://psytxjx.jxnu.edu.cn/oa/DArticle.aspx?type=view&id=202305012

[B37] Kashdan T. B. Barrett L. F. McKnight P. E. (2015). Unpacking emotion differentiation: transforming unpleasant experience by perceiving distinctions in negativity. Curr. Dir. Psychol. Sci. 24, 10–16. 10.1177/0963721414550708

[B38] Kienlen K. K. (1998). “*Developmental and social antecedents of stalking,” i*n *The Psychology of Stalking* (San Diego, CA: Academic Press), 51–67.

[B39] Knappe S. Beesdo-Baum K. Fehm L. Lieb R. Wittchen H. U. (2012). Characterizing the association between parenting and adolescent social phobia. J. Anxiety. Disord. 26, 608–616. 10.1016/j.janxdis.2012.02.014 22445318

[B40] Levy D. M. (1941). Maternal overprotection. Psychiatry 4, 567–626. 10.1080/00332747.1941.11022368

[B41] Li B. C. (2025). The doctrinal construction of the punishment scope of cyber violence crimes. Global L. Rev. 47, 5–23.

[B42] Li R. Xia L. X. (2021). The mediating effect of aggression motivation on the relationship between trait anger and reactive aggression: a longitudinal study. Acta Psychol. Sin. 53, 788–797. 10.3724/SP.J.1041.2021.00788

[B43] Li X. R. Duan X. H. Wang J. Z. Wu C. X. (2015). The relationships among parenting styles, adult attachment, and psychological distress of mongolian and han college students. J. Psychol. Sci. 38, 361–365. 10.16719/j.cnki.1671-6981.2015.02.036

[B44] Liu C. Ma J. L. (2019). Adult attachment style, emotion regulation, and social networking sites addiction. Front. Psychol. 10:2352. 10.3389/fpsyg.2019.02352 31749729 PMC6843004

[B45] Liu M. M. Guo F. Chen Z. Y. (2023). Effect of paternal overprotection on achievement motivation of high school students: chain mediation effect of autonomy and grit. Chin. J. Health Psychol. 31, 931–935. 10.13342/j.cnki.cjhp.2023.06.026

[B46] Liu Y. H. Liu Y. X. Wen J. H. (2022). Does anime, idol culture bring depression? Structural analysis and deep learning on subcultural identity and various psychological outcomes. Heliyon 8:e10567. 10.1016/j.heliyon.2022.e10567 36158100 PMC9489955

[B47] Lu D. (2018). A study on the involvement of idol worship in early adult fans and its relationship with self-efficacy and loneliness (Unpublished master's thesis). Yunnan Normal University, Kunming.

[B48] Luo Y. L. Zhang D. J. Liu Y. B. Liu Y. L. (2011). Reliability and validity of the Chinese version of trait anger scale in college students. Chin. Ment. Health J. 25,700–704. Available online at: https://kns.cnki.net/kcms2/article/abstract?v=7HNy6Ze5ODGrZivmgv0vyshOiX60ofdf8OeJLc-A-Tpb4AqHFLhsG0hO0RtYYjersbOyQuw1ntU2JCYPON645KU5oFyvzAwuXgmQ9gHUnm8b3_oI6zRqsSX5ft77WK1HxDM2EWodq1v4Ev7RD1ac9ZtoBTbGvgy7z2zpuCAnunbmznHcJRzyUg==&uniplatform=NZKPT&language=CHS

[B49] Marganska A. Gallagher M. Miranda R. (2013). Adult attachment, emotion dysregulation, and symptoms of depression and generalized anxiety disorder. Am. J. Orthopsychiat. 83, 131–141. 10.1111/ajop.12001 23330631

[B50] McCutcheon L. E. Lange R. Houran J. (2002). Conceptualization and measurement of celebrity worship. Br. J. Psychol. 93, 67–87. 10.1348/000712602162454 11839102

[B51] McCutcheon L. E. Scott Jr. V. B. Aruguete M. S. Parker J. (2006). Exploring the link between attachment and the inclination to obsess about or stalk celebrities. J. North Am. Psychol. 8, 289–300. Available online at: https://psycnet.apa.org/record/2006-09074-009

[B52] Mikulincer M. (1998a). Adult attachment style and individual differences in functional versus dysfunctional experiences of anger. J. Pers. Soc. Psychol. 74, 513–524. 10.1037/0022-3514.74.2.513 9491590

[B53] Mikulincer M. (1998b). Attachment working models and the sense of trust: an exploration of interaction goals and affect regulation. J. Pers. Soc. Psychol. 74, 1209–1224. 10.1037/0022-3514.74.5.1209

[B54] Miller J. G. Kahle S. Hastings P. D. (2015). Roots and benefits of costly giving: children who are more altruistic have greater autonomic flexibility and less family wealth. Psychol. Sci. 26, 1038–1045. 10.1177/0956797615578476 26015412 PMC4504814

[B55] Miller K. F. Borelli J. L. Margolin G. (2018). Parent-child attunement moderates the prospective link between parental overcontrol and adolescent adjustment. Fam. Process 57, 679–693. 10.1111/famp.12330 29057468 PMC8087184

[B56] Owens G. P. Held P. Hamrick L. Keller E. (2018). The indirect effects of emotion regulation on the association between attachment style, depression, and meaning made among undergraduates who experienced stressful events. Motiv. Emot. 42, 429–437. 10.1007/s11031-018-9688-0

[B57] Patchin J. W. Hinduja S. (Eds.). (2011). Cyberbullying Prevention and Response: Expert Perspectives, 1st Edn. Routledge. 10.4324/9780203818312

[B58] Peng W. B. Qiu X. T. Liu D. Z. Wang P. (2010). Revision of the scale of celebrity worship. Psy. Dev. Educ. 26, 543–548. 10.16187/j.cnki.issn1001-4918.2010.05.002

[B59] Peng X. Sun S. A. (2011). A brief discussion on the idol worship of contemporary college students. J. Liaoning Univ. Technol. 13, 95–96+99. Available online at: https://kns.cnki.net/nzkhtml/xmlRead/trialRead.html?dbCode=CJFD&tableName=CJFDTOTAL&fileName=LLGX201106031&fileSourceType=1&appId=KNS_BASIC_PSMC&invoice=hiFYBVnb26ZehPXmFl/re5NhuoxJgORGzEHmoDO9fGtXXueqrvEKIb2fWZwdRfG4+pb44EFlh1WVyMFhrq02ImZbyE4YUzNb4/DgkQCyjgVqJGhmqdQm7lds4P/ETE3i0mDkg3GOK26qjyd9vK11f2EL7P1KCnHcxrvJqSjUtQ4=

[B60] Pu X. T. Zhang A. B. (1996). Resistance to the socialization of young people's personalities. CN. Youth Stud. 8, 42–44. 10.19633/j.cnki.11-2579/d.1996.02.017

[B61] Qiao L. Z. Liu S. Cao S. J. Mu J. W. Xie S. S. Wang X. Y. (2023). Influence of parental rearing style on college students consumption behavior of fan economy: the chain mediating role of neryousness and interpersona problems. Chin. J. Health Psychol. 31, 1428–1434. 10.13342/j.cnki.cjhp.2023.09.027

[B62] Resick P. A. Monson C. M. Chard K. M. (2017). Cognitive Processing Therapy for PTSD: A Comprehensive Manual. The Guilford Press.

[B63] Rippere V. (1977). Commonsense beliefs about depression and antidepressive behaviour: a study of social consensus. Behav. Res. Ther. 15, 465–473. 10.1016/0005-7967(77)90002-X 603479

[B64] Ryan R. M. Deci E. L. Vansteenkiste M. (2016). “Autonomy and autonomy disturbances in self-development and psychopathology: Research on motivation, attachment, and clinical process,” in Developmental Psychopathology: Theory and Method, 3rd Edn., ed. D. Cicchetti (John Wiley & Sons, Inc.), 385–438.

[B65] Shi F. N. Fan Y. L. (2024). Analysis and countermeasures on college students' mental health issues based on spatiotemporal scale. Shanxi Youth 49, 94–97. Available online at: https://kns.cnki.net/kcms2/article/abstract?v=7HNy6Ze5ODHfoM7apXH7F5Q-7ImCxraxQdzAmQq_KF0OuNclpWc9HMyZGmYfpNVEfG2NSDMu7xJbxOpLrWJ1XfpiEhTb_CGZSpr6c_zLWTL5jX3c0GoIG4KZGC2UNLiEFW4cpCpU0_oMUygUs-9KxOyoyH9V2tJFoiKjCtyZC8TiQ5jG5GbeKQ==&uniplatform=NZKPT&language=CHS

[B66] Shi J. H. Zhang M. Y. Yang Y. Feng J. Kou Y. (2016). Celebrity worship and its relationship with aspirations and subjective well-being of junior middle school students: the moderating effect of gender. Psychol. Dev. Educ. 32, 666–674. 10.16187/j.cnki.issn1001-4918.2016.06.04

[B67] Shrout P. E. Bolger N. (2002). Mediation in experimental and nonexperimental studies: new procedure and recommendations. Psychol. Methods 7, 422–445. 10.1037/1082-989X.7.4.422 12530702

[B68] Song B. N. (2023). Anomie and reconstruction: social chaos in cyberspace and its governance strategies-taking “fandom culture” as an example. Hebei Acad. J. 43, 220–224. Available online at: https://kns.cnki.net/nzkhtml/xmlRead/trialRead.html?dbCode=CJFD&tableName=CJFDTOTAL&fileName=HEAR202303029&fileSourceType=1&appId=KNS_BASIC_PSMC&invoice=gDURdgQfxZIVviqHEyIehzSuurs+rYbwe0hisUepV0CjWox+s+wXnOKpm2BNAMLmY+7HLJOfYfTlldhVxs6uXfCJtvmT8PfuaFLsQOMS+SweUkZR3jJFGnIAfAD7Vwlx2KzfVgXQ2NayjUEBlMAW962/OaZPvs0qhYbEFY3pF6g=

[B69] Spielberger C. D. (1988). Manual for the State-Trait Anger Expression Inventory (STAXI). Odessa, FL: Psychological Assessment.

[B70] Strøm I. F. Thoresen S. Wentzel-Larsen T. Sagatun Å. Dyb G. (2014). A prospective study of the potential moderating role of social support in preventing marginalization among individuals exposed to bullying and abuse in junior high school. J. Youth Adolesc. 43, 1642–1657. 10.1007/s10964-014-0145-4 24985489 PMC4162984

[B71] Stroud C. B. Hershenberg R. Cardenas S. Greiter E. Richmond M. (2016). US college students' sexual activity: the unique and interactive effects of emotion regulation difficulties and attachment style. Int. J. Sex. Health 28, 37–49. 10.1080/19317611.2015.1073824

[B72] Sutton T. E. Simons L. G. (2021). A longitudinal test of a feminist pathways model among black youth: incorporating racial discrimination and school difficulties. Femin. Crim. 16, 26–46. 10.1177/1557085120923042

[B73] Tajfel H. (1978). Differentiation Between Social Groups: Studies in the Social Psychology of Intergroup Relations (chapters 1~*3)*. London: Academic Press.

[B74] Tang J. Y. Li X. Y. (2022). Relationship of parenting styles during covld-19 lsolation andbullying victimization and depression after returning to school. Chin. J. Clin. Psychol. 30, 377–381. 10.16128/j.cnki.1005-3611.2022.02.026

[B75] Tang Y. Y. Hölzel B. K. Posner M. I. (2015). The neuroscience of mindfulness meditation. Nat. Rev. Neurosci. 16, 213–225. 10.1038/nrn3916 25783612

[B76] Troisi A. D'Argenio A. (2004). The relationship between anger and depression in a clinical sample of young men: the role of insecure attachment. J. Affect. Disord. 79, 269–272. 10.1016/S0165-0327(02)00406-8 15023506

[B77] Valdez B. R. (2016). The effects of overprotective parenting on academic self-esteem: the moderating role of teachers (Unpublished master's thesis). West Virginia University, Morgantown, United States.

[B78] van der Voort A. Juffer F. Bakermans-Kranenburg M. (2014). Sensitive parenting is the foundation for secure attachment relationships and positive social-emotional development of children. J. Child Serv. 9, 165–176. 10.1108/JCS-12-2013-0038

[B79] Van Petegem S. Albert Sznitman G. Darwiche J. Zimmermann G. (2022). Putting parental overprotection into a family systems context: relations of overprotective parenting with perceived coparenting and adolescent anxiety. Fam. Process 61, 792–807. 10.1111/famp.12709 34435656

[B80] Van Petegem S. Antonietti J. P. Eira Nunes C. Kins E. Soenens B. (2020). The relationship between maternal overprotection, adolescent internalizing and externalizing problems, and psychological need frustration: a multi-informant study using response surface analysis. J. Youth Adolesc. 49, 162–177. 10.1007/s10964-019-01126-8 31583507

[B81] Vansteenkiste M. Lens W. Soenens B. Luyckx K. (2006). Autonomy and relatedness among Chinese sojourners and applicants: conflictual or independent predictors of well-being and adjustment? Motiv. Emot. 30, 273–282. 10.1007/s11031-006-9041-x

[B82] Wang P. Liu D. Z. (2010). A psychological exploration of adolescent idol worship. J. Soochow Univ. Philos. Soc. Sci. Educ. 49, 179–181. 10.19563/j.cnki.sdzs.2010.05.045

[B83] Wang X. C. Tan Y. H. (2012). “A discussion on the influence of campus idol culture on college ideological and political education-an analysis of cases in three universities in Dalian,” in Paper presented at the 5th Annual Conference on Education and Teaching Reform and Management Engineering (Chongqing, CN).

[B84] Wang Y. X. (2017). The legitimacy of paradox: the reproduction and production of fan images by online fan communities: a case study of Luhan's online fan community. CN. Youth Stud. 29, 67–74. 10.19633/j.cnki.11-2579/d.2017.06.011

[B85] Warren S. L. Bost K. K. Roisman G. I. Silton R. L. Spielberg J. M. Engels A. S.. (2010). Effects of adult attachment and emotional distractors on brain mechanisms of cognitive control. Psychol. Sci. 21, 1818–1826. 10.1177/0956797610388809 21098213 PMC3056541

[B86] Xu C. Yan W. H. (2023). Negative parenting styles and social adjustment of university students: a moderated chain mediation model. Curr. Psychol. 42, 27719–27732. 10.1007/s12144-022-03809-1 36340895 PMC9628611

[B87] Xu L. L. (2018). The influence of parenting pattern to subjective well-being of higher vocational college students: the mediating role of attributional style (Unpublished master's thesis). Hunan Normal University, Changsha, China.

[B88] Xu Y. L. Gao Z. H. Li J. M. (2009). Research on the relationship between parental rearing pattern and adult attachment of college students.Chin. J. Health Psychol. 17, 1228–1230. 10.13342/j.cnki.cjhp.2009.10.057

[B89] Yang Y. Q. Wu B. (2021). Relationship between idol worship and core self-evaluation of college students. J. Health Psychol. 29, 314–320. 10.13342/j.cnki.cjhp.2021.02.033

[B90] Yin H. X. (2004). Summary of domestic study on self-esteem. J. Harbin Univ. 25, 59–63. Available online at: https://kns.cnki.net/kcms2/article/abstract?v=7HNy6Ze5ODFVGHiz3P8D8PSowbOpbGtQBLJWAvztveH7RMdNPvcrxn-N4GM5n6tIFPbfuBNbMKkbW2imLjxmG-oG8BwWVF1YcecI1j8awuHWEqJxxPuqCZsKfuWlEF0o6iMaJlA1VEYRWVvkyXerhiut6ZHQNb3LNx7SGlfn0jUupmoYFkLjNA==&uniplatform=NZKPT&language=CHS

[B91] Yin Y. Y. (2020). An emergent algorithmic culture: the data-ization of online fandom in China. Int. J. Cultural Stud. 23, 475–492. 10.1177/1367877920908269

[B92] Yue D. M. Li M. Jin K. H. Ding B. K. (1993). Preliminary revision of EMBU and its application in neurotic patients. Chin. Ment. Health J. 7, 97–101. Available online at: https://kns.cnki.net/kcms2/article/abstract?v=7HNy6Ze5ODGPCFSmZkf9vOl0A11S50gOsdKY7neiboGj40gIqrmMZ1-McJbJNl9z–kOo7eH-FPhaU-zfanWfCvaPrXnDz-z_gMJD8pipCJFg6jIzNI2o576qFH4FAf2j8lO2NgmI0O4vBi_hVAK3bkU2Yfg5AHHTdUnQSWcXaJWHKHvsu2lmg==&uniplatform=NZKPT&language=CHS

[B93] Yue F. J. Yuan S. Xiao G. Y. Jiang X. H. (2024). Relationship between trait anger and mentalization of collegestudents: moderated mediation model. Chin. J. Health Psychol. 32, 314–320. 10.13342/j.cnki.cjhp.2024.02.028

[B94] Yue X. D. (2004). On idols - role model education. J. Chin. Soc. Educ. 137, 21–24+62. Available online at: https://kns.cnki.net/kcms2/article/abstract?v=7HNy6Ze5ODGdZ9aJCwD_audmxU9dQzBn4Libv6_gMUuzeGA-O_NVDYZpVJ9vEs92DVqUhb6GzC9KMtsT-3dzuomhprOxneUeUKjwYfBl14DjYBLDF1X_DDAv9BRKnrgnRsIolg8K9MlluFeiDj0WW_V-k8STk6GLS3ugNdFr6BpH58GfEHXtMQ==&uniplatform=NZKPT&language=CHS

[B95] Zhang B. J. Lv Y. (2010). Analyzing and countermeasuring about university students' ldolatry psychology. J. Jilin Norm. Univ. 38, 104–106. Available online at: https://kns.cnki.net/nzkhtml/xmlRead/trialRead.html?dbCode=CJFD&tableName=CJFDTOTAL&fileName=SLXS201003032&fileSourceType=1&appId=KNS_BASIC_PSMC&invoice=eTASLRzp/DbdvGwwYRQJJ3/7zWEaaQaV0bvjRXT+pnXnIDc/grCqPzjWrMPJe+pVPN0jkI3yVe/BqACJSpKYI6b9HRR2/xMIJnf6FNSHzTxWatBwzOyE6uO6Z7rQYA75Ym7XKbfqe1ilV8ytocppfwC94EncwKjx/kFdrkxadPw=

[B96] Zhang Q. Negus K. (2020). East Asian pop music idol production and the emergence of data fandom in China. Int. J. Cult. Stud. 23, 493–511. 10.1177/1367877920904064

[B97] Zhang Q. C. Li J. W. (2006). Relationship between the character of secondary school students' idolatry and parental rearing patterns. Chin. J. Health Psychol. 14, 610–612. 10.13342/j.cnki.cjhp.2006.06.004

[B98] Zhang R. S. (2012). Sandplay Therapy. Beijing: People's Education Press.

[B99] Zhang W. W. Lin L. Y. Xiong L. H. Li Y. Y. Lin X. H. He J. Y.. (2014). The relationship between adolescent violence and parenting styles. Chin. J. Sch. Health 35, 261–263. 10.16835/j.cnki.1000-9817.2014.02.038

[B100] Zhang Y. R. Zuo B. (2006). Social identity theory and it's development. Adv. Psychol. Sci. 14, 475–480. Available online at: https://journal.psych.ac.cn/adps/EN/abstract/abstract2618.shtml

[B101] Zhou Z. K. Liu Q. X. (2016). Cyber-psychology: the reconstruction of behavior. Chin. Soc. Sci. Rev, 1, 55–67+126–127. Available online at: https://kns.cnki.net/reader/flowpdf?invoice=ByEs59jVNsCR9sTVcJYZ%2Fu9FB8uhbfTihy8kbi0kUVkGITy5VIJn4lZK0XiWZSRXhVA2DHgTelGNyRTdyiIk0i91uPA7CgPj7O2neEtSc%2FdaNbqhiKBZx2UKXRRPEIF7OjGE1dZh7kF3M24mT%2Bzyh0sp5lT%2BxiCPPVCr5FoxMrA%3D&platform=NZKPT&product=CJFQ&filename=ZSKP201603005&tablename=cjfdlast2016&type=JOURNAL&scope=trial&dflag=pdf&pages=&language=CHS&trial=&nonce=B8D7CF5ABAE14327B9DC231BCF1D93AA&cflag=pdf

[B102] Zhu L. T. Liu Y. L. (2003). A study of personality dysfunction and relevant factors. J. Nanjing Univ. Aero. Astro. 5, 82–86. Available online at: https://kns.cnki.net/kcms2/article/abstract?v=7HNy6Ze5ODHGivb2akNV51PVEToPTnTd0R5PlX62N8f0W_3QTB7ypK6V1UXawHIi7Vsj9wPnMt9YzyVm8sqq6eFMKzy4bMEux9iUv4_EQBOMcFnAgvxnoNZYAlFMBy_1X7zYiEeQrQWpGrxnA5teI5eIAquoEIeKSiMm2EScHUqxpUhli7mP-w==&uniplatform=NZKPT&language=CHS

